# The Effect of Arterial Elongation on Isolated Common Iliac Artery Pathologies

**DOI:** 10.3390/life14111440

**Published:** 2024-11-07

**Authors:** Ádám Szőnyi, Balázs Bence Nyárády, Márton Philippovich, Adrienn Dobai, Ekrem Anil Sari, András Szőnyi, Anikó Ilona Nagy, Edit Dósa

**Affiliations:** 1 Heart and Vascular Center, Semmelweis University, 1122 Budapest, Hungary; szonyi.adam@stud.semmelweis.hu (Á.S.);; 2 Department of Oral Diagnostics, Semmelweis University, 1088 Budapest, Hungary; 3 Dokuz Eylül University Research and Application Hospital, 35330 Izmir, Turkey; ekremanil.sari@deu.edu.tr; 4 Gottsegen National Cardiovascular Center, 1096 Budapest, Hungary

**Keywords:** aorta, iliac arteries, elongation, tortuosity, curvature, stenosis, occlusion, aneurysm

## Abstract

Purpose: to investigate the effects of vessel geometry on steno-occlusive and dilatative common iliac artery (CIA) pathologies. Methods: this single-center, retrospective study included 100 participants, namely 60 participants with a unilateral, isolated CIA pathology who were divided into three pathology-based groups (a stenosis group, *n* = 20, an occlusion group, *n* = 20, and an aneurysm group, *n* = 20) and 40 participants without a CIA pathology (control group). All participants underwent abdominal and pelvic computed tomography angiography. The aortoiliac region of the participants was reconstructed into three-dimensional models. Elongation parameters (tortuosity index (TI) and absolute average curvature (AAC)) and bifurcation parameters (iliac take-off angle, iliac planarity angle, and bifurcation angle) were determined using an in-house-written piece of software. Demographic data, anthropometric data, cardiovascular risk factor data, and medical history data were obtained from participants’ electronic health records. The following statistical methods were used: one-way ANOVA, chi-square test, *t*-tests, Wilcoxon test, Kruskal–Wallis test, and multivariate linear regression. Results: in the occlusion group, both TI and AAC values were significantly higher on the contralateral side than on the ipsilateral side (both *p* < 0.001), whereas in the aneurysm group the AAC values were significantly higher on the ipsilateral side than on the contralateral side (*p* = 0.001). The ipsilateral and contralateral TI and AAC values of the iliac arteries were significantly higher in the aneurysm group than in the other three groups (all *p* < 0.001). Age significantly affected all of the elongation parameters except for the TI of the infrarenal aorta (all *p* < 0.010 except the TI of the infrarenal aorta). In addition, the AAC values for the iliac arteries were significantly associated with obesity (ipsilateral iliac artery, *p* = 0.045; contralateral iliac artery, *p* = 0.047). Aortic bifurcation parameters did not differ significantly either within each group (ipsilateral versus contralateral side) or between the individual groups. Conclusions: occlusions tend to develop in relatively straight iliac arteries, whereas unilateral, isolated CIA aneurysms are more likely to occur in elongated aortoiliac systems.

## 1. Introduction

Lower extremity arterial disease (LEAD), which can be occlusive or dilatative, affects approximately 230 million people worldwide. Approximately 20% of people over the age of 80 have LEAD [1,2], and 25–31% of LEAD cases are localized to the aortoiliac region [3,4,5]. The aortoiliac steno-occlusive disease can cause mild-to-severe discomfort or significant pain in the buttocks and lower limbs, and it delays wound healing and can lead to minor or major amputation in patients with irreversible tissue damage. In addition, the aortoiliac disease can result in erectile dysfunction. Similarly to steno-occlusive arterial diseases, dilatative arterial diseases can cause acute or chronic buttock and lower limb pain, but the greatest danger of dilated arterial diseases is that arteries can rupture and produce life-threatening bleeding. Within aortoiliac stenoses/occlusions, the incidence of isolated common iliac artery (CIA) stenoses or occlusions is 25–30% [6]. Although isolated CIA aneurysms are relatively rare, one study estimates that these aneurysms account for 25% of all iliac branch device (IBD) implantations [7]. Various parameters such as cardiovascular (CV) risk factors, geometric features, and lesion characteristics have been shown to be associated with the development of CIA steno-occlusive or dilatative diseases [8,9]. However, we still have limited knowledge of the effects of iliac artery elongation on CIA pathologies. Most previous research has focused on the effects of iliac artery elongation in the context of endovascular aneurysm repair and IBD procedures and has found that increased iliac artery elongation is linked to a higher risk of complications, particularly endoleaks [10,11]. Other studies have looked at the elongation of the iliofemoral tract in relation to access site complications (e.g., bleeding) that occur during or after endovascular interventions [12,13]. Nevertheless, there are still gaps in our understanding of how iliac artery elongation influences CIA pathologies. The aim of this study is to describe the geometric properties of the aortoiliac region in patients diagnosed with steno-occlusive or dilatative CIA pathology.

## 2. Materials and Methods

### 2.1. Selection of Participants

This single-center, retrospective study was conducted in a cohort of 100 participants at the Heart and Vascular Center of Semmelweis University between 2012 and 2024. Sixty patients were enrolled consecutively into three groups of 20 based on CIA pathology: a stenosis group, an occlusion group, and an aneurysm group. In addition, 40 participants without aortoiliac pathology were included in the control group. The control group encompassed individuals aged between 50 and 80 years so that the male-to-female ratio was as similar as possible to the male-to-female ratio of the other groups. All participants underwent abdominal and pelvic computed tomography angiography (CTA; Philips Brilliance iCT 256, Philips Medical Systems International B.V., Best, The Netherlands).

Patients with unilateral, isolated, significant CIA stenosis were assigned to the stenosis group, whereas patients with unilateral complete CIA occlusion were assigned to the occlusion group. Significant stenosis was defined as a 70–99% reduction in the arterial lumen. Exclusion criteria in relation to both groups consisted of having a stenosis grade greater than 50% anywhere else in the aortoiliac region or any dilatative pathology of the infrarenal aorta or iliac arteries. Lesion characteristics such as lesion location, grade of stenosis, lesion length, and degree of calcification were recorded in both groups. The grade of stenosis was calculated using the following formula:

$$stenosis\;\left(\%\right)=\left(\frac{normal\;diamter-stenotic\;diameter}{normal\;diameter}\right)\times 100$$

Vessel diameter, lesion length, and degree of calcification were determined on multiplanar and curved planar reconstructed CTA images. The degree of calcification of steno-occlusive lesions was assessed using the method developed by Huynh et al. [14]. In this system, the total calcification score consists of a morphology score (appearance of calcification; score range: 0–3 points), a circumference score (percentage of circumference affected; score range: 0–4 points), and a length score (percentage of length involved; score range: 0–4 points). With a score of 0–7, the lesion is considered mildly calcified, with a score of 8–9, the lesion is considered moderately calcified, and, with a score of 10–11, the lesion is considered severely calcified [14].

The aneurysm group consisted of patients with unilateral, isolated CIA aneurysm (Reber type I) [15]. According to the guidelines of the European Society for Vascular Surgery, an aneurysm is defined as a 1.5-fold increase in the normal CIA diameter [16]. Exclusion criteria comprised having steno-occlusive disease in any iliac artery, having an abdominal aortic aneurysm, or having a bilateral CIA aneurysm. Lesion characteristics such as aneurysm location and largest aneurysm diameter were collected.

Data describing demographic characteristics, anthropometric parameters, CV risk factors, and the medical history of the study participants were extracted from electronic health records. The data collected for CV risk factors included each participants’ age, sex, and smoking status in addition to the presence or absence of each of the following conditions: obesity, hypertension, diabetes mellitus, hyperlipidemia, and chronic kidney disease. The definition of CV risk factors can be found in the publication by Dósa et al. [17]. This study was conducted in accordance with the Declaration of Helsinki and was approved by the Semmelweis University Regional and Institutional Committee of Science and Research Ethics (approval number: 4/2024). As this study accessed only fully anonymized data, the need to obtain informed consent from participants was waived by the aforementioned committee.

### 2.2. Image Processing

**Figure 1:**
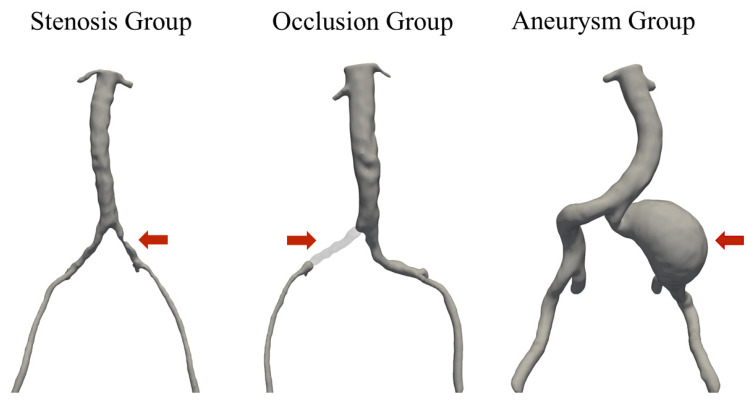
Reconstructed three-dimensional model of common iliac artery stenosis, occlusion, and aneurysm.

Computed tomography angiography images of all participants were imported into the 3D Slicer software (Slicer 3D 2024. Version 5.6.2.) [18,19,20,21,22] to perform segmentation of images of the aortoiliac region (Figure 1). The segmented models were then exported and analyzed using the Python software that was written in-house based on the vascular modeling toolkit (VMTK) [23,24]. Several studies have confirmed the reliability and accuracy of VMTK-generated measurements [25,26]. The evaluation that was performed for each participant included the extraction of centerlines, the calculation of arterial elongation, and the assessment of aortic bifurcation geometry.

**Figure 2:**
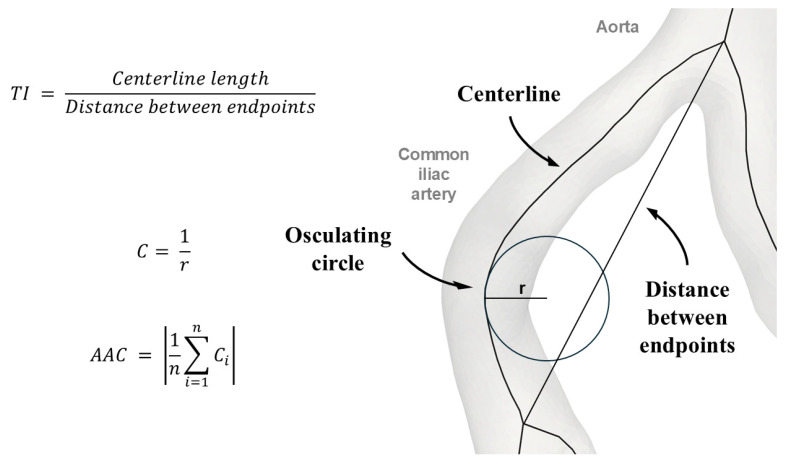
Schematic representation of the calculation of the tortuosity index and the absolute average curvature. AAC—absolute average curvature; C—curvature; C_i_—each curvature measurement, where i is an index from 1 to *n*; *n*—the total number of curvature measurements; *r*—radius; TI—tortuosity index.

Centerlines were generated for the infrarenal aorta and the left and right iliac arteries (the common and external iliac arteries). Arterial elongation was quantified using two metrics: the tortuosity index (TI) and the absolute average curvature (AAC). The TI was computed by dividing the length of the centerline by the shortest distance between its endpoints. The AAC was calculated by sampling the reciprocal of the radius of osculating circles at 0.5 cm intervals and by averaging these values (Figure 2). A vector system was created to obtain the geometry of the aortic bifurcation. In-plane vector orientations were used to determine CIA take-off angles and aortic bifurcation angles. In addition, out-of-plane vector orientations were used to derive the planarity of CIAs, that is, their anteroposterior planar inclination relative to the aorta. The segmentation and elongation measurement procedures are described in further detail in our research group’s previous publication [27].

### 2.3. Statistical Analysis

Continuous variables were presented as medians and interquartile ranges (IQRs). Categorical variables were presented as numerical and percentage values. Demographic data, anthropometric parameters, and CV risk factors were compared using one-way ANOVA tests or chi-square tests. The lesion length was compared between the stenosis and occlusion groups using an independent *t*-test, while the degree of calcification was compared using the chi-square test. Paired *t*-tests and Wilcoxon tests were used to determine the differences among the TI, the AAC, and the take-off and planarity angles of the ipsilateral and contralateral iliac arteries. Kruskal–Wallis tests with pairwise comparisons and one-way ANOVA tests with Tukey or Games–Howell post hoc tests (depending on the homogeneity of variance) were performed to examine differences among the groups (the stenosis group, the occlusion group, the aneurysm group, and the control group) in the TI, the AAC, and the aortic bifurcation metrics. To assess the various relationships of age, sex, CV risk factors, and patient group with aortoiliac morphology, multivariate linear regression models using stepwise selection were created and included the TI, AAC, and take-off and planarity angles as dependent variables. Statistical analyses were conducted using IBM SPSS 28 (SPSS Inc., Chicago, IL, USA), and statistical significance was defined at the *p* < 0.05 level.

## 3. Results

### 3.1. Patient Demographics, Anthropometric Data, and Cardiovascular Risk Factors

There were significant differences among the groups with respect to male sex (*p* = 0.035), weight (*p* = 0.006), height (*p* = 0.033), body mass index (*p* = 0.021), body surface area (*p* = 0.005), and diabetes mellitus (*p* = 0.008). The incidence of male sex was highest in the aneurysm group (95%). Those in the aneurysm group had the highest median weight (88 kg (80.8–97.3 kg)) and median height (176 cm (169.5–180 cm)). Diabetes mellitus was least common among patients with CIA aneurysms (5%) (Table 1).

### 3.2. Lesion Characteristics

In the stenosis group, the left CIA was affected in 13 patients (65%), whereas the right CIA was affected in seven patients (35%). In the occlusion group, the left CIA was affected in eight patients (40%), whereas the right CIA was affected in 12 patients (60%). The lesions in the occlusion group were significantly longer than in the stenosis group (*p* = 0.006), while there was no significant difference in the grade of calcification between the stenosis and occlusion groups (*p* = 0.860). Most patients’ aneurysms were located in the right CIA (*n* = 14, 70%), whereas the left CIA was affected in six patients (30%) (Table 1).

### 3.3. Arterial Elongation

In the stenosis group, there were no significant differences in the elongation parameters between the ipsilateral and contralateral iliac arteries. In the occlusion group, both the TI and AAC values were significantly higher on the contralateral side than on the ipsilateral side (TI, *p* < 0.001; AAC, *p* < 0.001). In the aneurysm group, the AAC values were significantly higher on the ipsilateral side than on the contralateral side (*p* = 0.001). The results of the arterial elongation analyses are outlined in Table 2.

**Figure 3:**
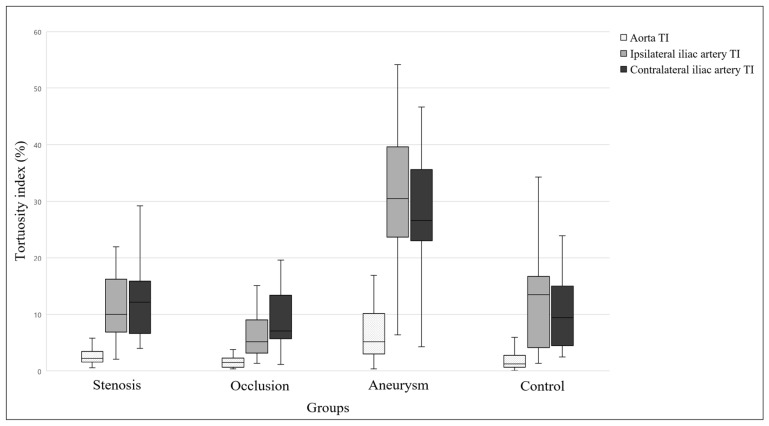
Results of the tortuosity index comparison. The graph shows the median, interquartile range, minimum, and maximum of the data. TI—tortuosity index.

**Figure 4:**
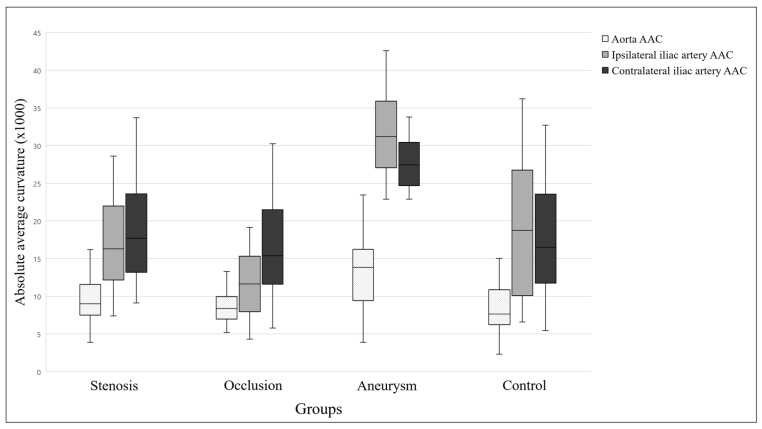
Results of the absolute average curvature comparison. The graph shows the median, interquartile range, minimum, and maximum of the data. AAC—absolute average curvature.

Significant differences were found among the groups for all of the TI parameters (all *p* < 0.001). The TI value for the infrarenal aorta was significantly higher in the aneurysm group than in the control and occlusion groups (both *p* < 0.001). In addition, the ipsilateral and contralateral TI values of the iliac arteries were significantly higher in the aneurysm group than in the other three groups (all *p* < 0.001). Similarly, AAC values also differed significantly among the groups. The AAC value of the infrarenal aorta was significantly higher in the aneurysm group than in the control and occlusion groups (both *p* < 0.001). Furthermore, the ipsilateral and contralateral AAC values of the iliac arteries were significantly higher in the aneurysm group than in the other three groups (all *p* < 0.001). A pairwise comparison of the elongation parameters is shown in Figure 3 and Figure 4.

Elongation parameters differed significantly based on patient group (all *p* < 0.001). Age was also significantly associated with all elongation parameters except for the TI of the infrarenal aorta (all *p* < 0.010 except for the TI of the infrarenal aorta). In addition, the AAC values for the iliac arteries differed significantly based on the presence of obesity (ipsilateral iliac artery, *p* = 0.045; contralateral iliac artery, *p* = 0.047). The results of the multivariate linear regression analysis are detailed in Table 3.

### 3.4. Aortic Bifurcation Geometry

The median aortic bifurcation angle was 65.4° (IQR, 57.4–70°) in the stenosis group, 59.5° (IQR, 52.4–61.6°) in the occlusion group, 59.9° (IQR, 46.5–75.1°) in the aneurysm group, and 53.1° (IQR, 45.3–60.8°) in the control group. No significant differences in iliac take-off and planarity angles between the ipsilateral and the contralateral side were detected in any of the groups (Table 4).

**Figure 5:**
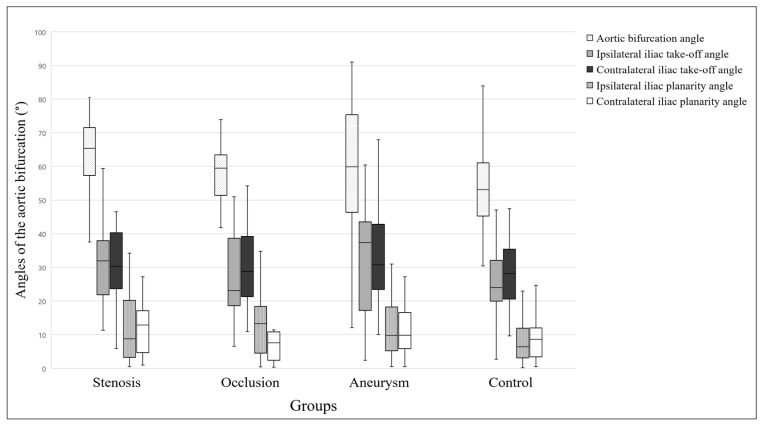
Comparison of aortic bifurcation parameters. The graph shows the median, interquartile range, minimum, and maximum of the data.

A groupwise analysis demonstrated no significant differences in aortic bifurcation angle, iliac take-off angles, and iliac planarity angles among the groups (Figure 5).

Cardiovascular risk factors showed no significant associations with either the aortic bifurcation angle or the iliac planarity angles. The ipsilateral iliac take-off angles differed significantly depending on the presence of diabetes mellitus (b = −0.19, *p* = 0.048), whereas the contralateral iliac take-off angles differed significantly based on patient group (b = −0.25, *p* = 0.009) and smoking status (b = −0.28, *p* = 0.004).

## 4. Discussion

In our study, we observed a male-to-female ratio of 19:1 in patients with isolated CIA aneurysms. A similar male predominance was reported by Fargion et al. [7], who analyzed 231 patients with isolated CIA aneurysms, of whom 218 (94.4%) were male.

Only one patient in our aneurysm group had diabetes mellitus. This result is not surprising, since it has been previously revealed that the development of abdominal aortic aneurysms is inversely associated with the presence of diabetes mellitus [28,29]. We assume that the reason for this inverse correlation may be the same for CIA aneurysms.

**Figure 6:**
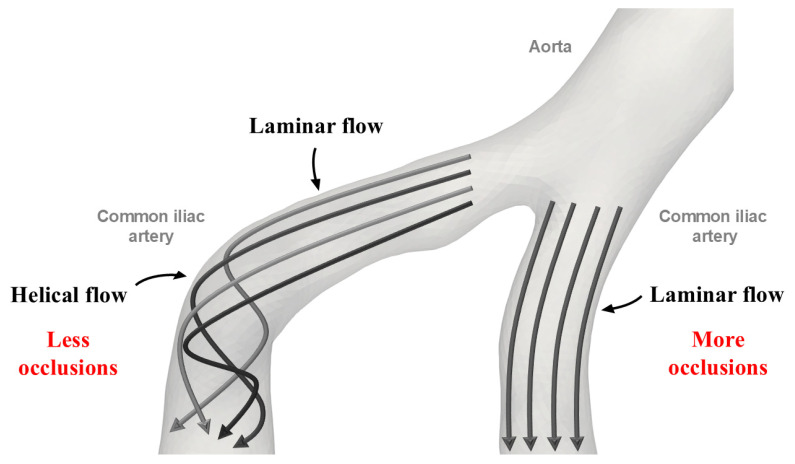
Schematic representation of the arterial secondary (helical) flow.

We found, in our study, that contralateral iliac artery elongation was greater than ipsilateral (with pathology) iliac artery elongation in patients with isolated CIA occlusion. This result seems surprising given the known proatherogenic effects of increased curvature and tortuosity which include altered wall shear stress. Therefore, we expected that the elongation of the ipsilateral iliac artery, but not the contralateral iliac artery, would be more pronounced in the occlusion group. A possible explanation for our observation could be arterial secondary flow. Arterial blood flow typically consists of primary (laminar) and secondary flow components (Figure 6). Centrifugal and torsional forces generate the secondary flow components in curves and branch points [30,31]. Helical secondary flow components have been detected in arterial bends [32], and several studies have shown that these helical flows can enhance blood transport, reduce blood cell adhesion, and prevent the accumulation of atherogenic low-density lipoproteins [33,34]. The atheroprotective and flow-stabilizing properties of helical flow have been documented in many segments of the human arterial system such as the coronary arteries, the carotid bifurcation, and the aorta [35,36,37]. However, further studies are needed to evaluate the flow characteristics of the aortoiliac region in the presence of steno-occlusive disease.

In our aneurysm group, significantly higher AAC values were noted in the ipsilateral (with pathology) iliac artery compared to the contralateral iliac artery. In addition, significantly higher elongation values were observed in the aneurysm group compared to the other groups (the stenosis group, the occlusion group, and the control group). The underlying hemodynamic mechanism for these observations may be related to wall shear stress. Parker et al. [38] examined 25 isolated CIA aneurysms in 23 patients; they created a three-dimensional reconstruction of the infrarenal aorta and CIAs, measured CIA elongation and aortic bifurcation parameters, and performed computational fluid dynamics (CFD) simulations. Although no comparison was made between the tortuosity values of the ipsilateral and the contralateral iliac arteries, a significantly lower time-averaged wall shear stress was revealed on the ipsilateral side. In a simulation study, Lu and Zhang [39] constructed artificial models of the right coronary artery with varying degrees of arterial bends to mimic different curvatures. They demonstrated that increased arterial tortuosity leads to areas of low wall shear stress. Changes in arterial wall shear stress have been associated with the synthesis and secretion of nitric oxide, growth factors, and metalloproteins [40]. These alterations in shear stress and the consequent mechanical and molecular effects may explain why CIA aneurysms occur more frequently in the elongated iliac artery. In addition, a study examining changes in abdominal aortic diameter over time has shown that increased tortuosity predisposes to dilatation and aneurysm formation [41]. This supports the idea that alterations in arterial geometry, particularly increased curvature, create regions that are more susceptible to aneurysm development. A greater increase in aneurysm size in areas of increased curvature also suggests that curvature is more likely a contributor to aneurysm formation rather than a consequence of it [41]. Studies also indicate that elastin in the extracellular matrix acts as the primary load-bearing component against both circumferential and longitudinal forces. When elastin weakens or fails, the vessel becomes more susceptible to elongation and aneurysm formation. Under normal blood pressure, elongated arteries develop areas influenced by local forces that predispose them to aneurysm formation (focal dilation), while elevated blood pressure increases the effect of circumferential forces, causing diffuse dilation of the vessels. This latter dilation amplifies the longitudinal forces, promoting further elongation of the artery. Overall, elastin appears to play a key role in arterial elongation and aneurysm development, and both conditions have the potential to contribute to the formation of the other [42,43]. However, further research is needed to fully understand the process of aneurysm development.

In our previous publication, we identified age as a significant factor influencing the elongation of the infrarenal aorta and iliac arteries [27]. The current study shows a similar pattern, with age significantly impacting TI and AAC values in both the infrarenal aorta and the ipsilateral and contralateral iliac arteries. According to the literature, the amount of collagen fibers in the arterial wall increases with age, causing the formation of cross-links and adhesions which, in turn, may contribute to the elongation of the arteries [44].

We also found that obesity significantly affects both ipsilateral and contralateral AAC values. Obesity has been linked to arterial elongation in various regions of the arterial system, including the internal carotid artery [45] and the thoracic aorta [46]. However, the exact mechanism by which obesity elongates the iliac arteries remains unclear. One hypothesis is that increased visceral fat leads to higher intra-abdominal pressure. This pressure may generate static forces in the abdominal cavity that act as a mechanical factor to stretch the iliac arteries [45,47].

Our results also suggest that iliac take-off angles are significantly reduced on the ipsilateral side in patients with diabetes mellitus and on the contralateral side in smokers compared to other patients. However, we cannot provide a meaningful explanation for this observation.

Our study has certain limitations such as its retrospective, single-center design and the small number of subjects per group. Due to the retrospective nature of our study, there is a risk of selection bias and no true causality can be established.

In conclusion, occlusions tend to develop in minimally curved iliac arteries, whereas unilateral, isolated CIA aneurysms are more likely to occur in elongated aortoiliac systems. Our study may also have clinical significance. For example, in addition to the traditional CV risk factors, anatomical features may sometimes be worth considering as potential predisposing factors for the development of peripheral artery disease. However, to better understand these dynamics, CFD studies should be conducted to gain more detailed insights into the blood flow patterns of steno-occlusive and dilatative CIA pathologies.

## Figures and Tables

**Table 1:** Age, gender, anthropometric data, cardiovascular risk factors, and lesion characteristics.

Parameters	Stenosis Group (*n* = 20)	Occlusion Group (*n* = 20)	Aneurysm Group (*n* = 20)	Control Group (*n* = 40)	*p*-Value
**Age (years),** **median (IQR)**	67 (64.8–76)	65 (61–70.8)	70.5 (62.8–78.3)	66.5 (60.8–71.3)	0.076
**Male sex, *n* (%)**	15 (75)	11 (55)	19 (95)	27 (67.5)	0.035
**Anthropometric data,** **median (IQR)**					
Weight (kg)	75 (69.5–87.5)	73.5 (64.8–85.3)	88 (80.8–97.3)	76.5 (68–86)	0.006
Height (cm)	172 (166.5–174.3)	167.5 (163.5–174.3)	176 (169.5–180)	170 (164.3–175)	0.033
BMI (kg/m^2^)	25.5 (23.5–28.6)	26.2 (23.5–28.6)	29.7 (26.3–32. 9)	27.2 (26.3–30.7)	0.021
BSA (m^2^)	1.9 (1.8–2.1)	1.9 (1.7–2)	2 (2–2.2)	1.9 (1.8–2)	0.005
**CV risk factors,** ***n* (%)**					
Obesity	4 (20)	3 (15)	9 (45)	14 (35)	0.126
Smoking	9 (45)	9 (45)	8 (40)	10 (25)	0.302
Hypertension	17 (85)	16 (80)	16 (80)	34 (85)	0.935
Diabetes mellitus	8 (40)	4 (20)	1 (5)	18 (45)	0.008
Hyperlipidemia	17 (85)	17 (85)	15 (75)	28 (70%)	0.458
CKD	1 (5)	0 (0)	1 (5)	3 (7.5%)	0.664
**Lesion** **characteristics**					
Stenosis grade (%), median (IQR)	75 (70–80)	100 (100–100)	NA	NA	<0.001
Lesion length (mm), median (IQR)	16.5 (12.8–25)	47 (39–53.3)	NA	NA	0.006
Mild calcification, *n* (%)	7 (35)	9 (45)	NA	NA	0.605
Moderate calcification, *n* (%)	8 (40)	6 (30)	NA	NA	0.651
Severe calcification, *n* (%)	5 (25)	5 (25)	NA	NA	0.925
Diameter (mm), median (IQR)	NA	NA	37 (33.5–47.3)	NA	NA

BMI—body mass index; BSA—body surface area; CKD—chronic kidney disease; CV—cardiovascular; IQR—interquartile range; NA—not applicable.

**Table 2:** Results of ipsilateral versus contralateral iliac artery elongation.

**TI (%),** **median (IQR)**	**Ipsilateral iliac artery (with pathology)**	**Contralateral iliac artery (without pathology)**	***p*-Value**
Stenosis group	9.97 (6.89–15.67)	12.14 (7.12–15.87)	0.296
Occlusion group	5.12 (3.46–8.63)	7.01 (5.76–13.31)	<0.001
Aneurysm group	30.48 (24.15–39.56)	26.58 (23.2–35.59)	0.668
	**Right iliac artery** **(without pathology)**	**Left iliac artery** **(without pathology)**	***p*-Value**
Control group	13.49 (4.26–16.18)	9.42 (4.75–14.68)	0.232
**AAC (×1000),** **median (IQR)**	**Ipsilateral iliac artery (with pathology)**	**Contralateral iliac artery (without pathology)**	***p*-Value**
Stenosis group	16.3 (12.25–20.73)	17.7 (13.33–22)	0.076
Occlusion group	11.65 (8.65–14.91)	15.4 (12.2–20.7)	<0.001
Aneurysm group	31.2 (27.34–35.19)	27.44 (25.43–30.28)	0.001
	**Right iliac artery** **(without pathology)**	**Left iliac artery** **(without pathology)**	***p*-Value**
Control group	18.77 (10.13–26.64)	16.51 (11.83–22.83)	0.160

AAC—absolute average curvature; IQR—interquartile range; TI—tortuosity index.

**Table 3:** Multivariate linear regression analysis of the elongation parameters.

Dependent Variables	Independent Variables	Beta	CI for Beta		*p*-Value
			Lower Bound	Upper Bound	
**TI—infrarenal aorta**	Patient group	−0.36	−0.011	−0.03	<0.001
**TI—ipsilateral iliac artery**	Patient group	−0.49	−0.05	−0.16	<0.001
	Age	0.38	0.002	0.007	<0.001
**TI—contralateral iliac artery**	Patient group	−0.36	−0.043	−0.015	<0.001
	Age	0.4	0.003	0.006	<0.001
**AAC—infrarenal aorta**	Patient group	−0.33	−0.002	−0.001	<0.001
	Age	0.26	0.000	0.001	0.007
**AAC—ipsilateral iliac artery**	Patient group	−0.3	−0.004	−0.001	<0.001
	Age	0.42	0.000	0.001	<0.001
	Obesity	0.17	0.000	0.007	0.045
**AAC—contralateral iliac artery**	Patient group	−0.29	−0.003	−0.001	<0.001
	Age	0.42	0.000	0.001	<0.001
	Obesity	0.17	0.000	0.006	0.047

AAC—absolute average curvature; CI—confidence interval; TI—tortuosity index.

**Table 4:** Ipsilateral versus contralateral iliac artery angles.

**Iliac take-off angles (°),** **median (IQR)**	**Ipsilateral side** **(with pathology)**	**Contralateral side** **(without pathology)**	***p*-Value**
Stenosis group	31.92 (24.46–37.81)	30.35 (24.17–39.95)	0.819
Occlusion group	23.13 (18.84–36.92)	28.79 (22.43–38.51)	0.413
Aneurysm group	37.3 (17.31–42.78)	30.75 (23.75–41.8)	0.816
	**Right side** **(without pathology)**	**Left side** **(without pathology)**	***p*-Value**
Control Group	23.98 (20.36–32.04)	28.11 (22.13–35.36)	0.565
**Iliac planarity angles (°),** **median (IQR)**	**Ipsilateral side** **(with pathology)**	**Contralateral side** **(without pathology)**	***p*-Value**
Stenosis group	8.77 (3.25–19.82)	12.95 (6.11–16.94)	0.117
Occlusion group	13.34 (5.46–17.71)	7.63 (2.42–10.77)	0.940
Aneurysm group	9.77 (6.12–17.1)	9.83 (6–15.6)	0.765
	**Right side** **(without pathology)**	**Left side** **(without pathology)**	***p*-Value**
Control group	6.42 (3.53–11.37)	8.6 (3.69–11.77)	0.436

IQR—interquartile range.

## Data Availability

The data presented in this study are available upon request from the corresponding author. The data are not publicly available due to reasons pertaining to patient privacy.
